# Exploring the Relationships Between Rehabilitation and Survivors of Intimate Partner Violence: A Scoping Review

**DOI:** 10.1177/15248380231196807

**Published:** 2023-09-30

**Authors:** Danielle Toccalino, Gifty Asare, Jenna Fleming, Joyce Yin, Amy Kieftenburg, Amy Moore, Halina (Lin) Haag, Vincy Chan, Jessica Babineau, Nneka MacGregor, Angela Colantonio

**Affiliations:** 1 University of Toronto, ON, Canada; 2 Centre for Addiction and Mental Health, Toronto, ON, Canada; 3 Eden Dales Social Work and Counselling Services, Toronto, ON, Canada; 4 Wilfrid Laurier University, Waterloo, ON, Canada; 5 National Service Dogs, Kitchener, ON, Canada; 6 University Health Network, Toronto, ON, Canada; 7 Women’s Centre for Social Justice, Toronto, ON, Canada

**Keywords:** domestic violence, intervention/treatment, battered women

## Abstract

Intimate partner violence (IPV) is a public health crisis affecting one in three women and one in ten men in their lifetimes. Rehabilitation professionals are highly likely to encounter survivors of IPV in their practice; yet, there exists no formal review assessing the relationship between IPV and rehabilitation. Our objective was to understand the types and contexts of rehabilitation care currently available for survivors of IPV, opportunities identified in the literature for rehabilitation care, and IPV awareness and education among rehabilitation providers. A search strategy related to IPV and four rehabilitation professionals of interest (occupational therapy, physiotherapy, speech-language pathology/therapy, and physiatry) was developed across 10 databases and complemented by a gray literature search. Two reviewers independently assessed articles for inclusion. In all, 44 articles met inclusion criteria, ranging from primary research articles (48%) to clinical newsletters. Included articles predominantly focused on opportunities for rehabilitation care (68%) and occupational therapists as a profession (68%). A minority of studies examined specific interventions for IPV survivors (18%) or assessed for knowledge and attitudes about IPV (16%) among rehabilitation professionals. To our knowledge, this is the first scoping review exploring the rehabilitation literature for IPV survivors. These findings show an awareness of IPV among rehabilitation professionals, the importance of identifying IPV in clients, and the ways in which rehabilitation professionals are uniquely situated to support survivors of IPV. There remains an opportunity to explore interventions designed specifically for IPV survivors.

## Background and Rationale

Intimate partner violence (IPV) is a highly prevalent public health concern that impacts one in three women and nearly one in ten men in their lifetime (Cotter, 2021; Smith et al., 2018; World Health Organization, 2021b). Defined as physical, psychological, sexual, emotional, or financial violence or abuse committed by a current or former spouse or intimate partner (Johnson et al., 2022; King et al., 2017; World Health Organization, 2021b), IPV can have significant impacts on the physical, mental, and emotional well-being of survivors, which may result in longer-term disability (Beydoun et al., 2017; Burczycka, 2018; Kwako et al., 2011; Stubbs & Szoeke, 2022). In addition to IPV increasing the risk of disability, individuals living with disabilities are at higher risk of IPV. Estimates suggest more than half of women living with disabilities experience some form of IPV in their lifetime and women living with disabilities experience almost double the rate of physical violence than women living without disabilities (Chirwa et al., 2020; Savage, 2021).

Due to both the risk of physical injury accompanying IPV and the high rate of IPV among individuals living with disability, IPV survivors are likely to benefit from rehabilitation. Rehabilitation refers to individualized interventions designed to reduce the impact of health conditions and support individuals in being “as independent as possible in everyday activities,” with interventions often including modifying living or working environments, adapting tasks, or implementing assistive supports (World Health Organization, 2021a). As such, rehabilitation professionals—such as physiotherapists, occupational therapists, speech-language pathologists/therapists, physiatrists/physical medicine, and rehabilitation physicians—are highly likely to encounter IPV survivors in their practice. Though related, each of these professions has specific aims. Occupational therapists (OTs), providing occupational therapy (OT), are “regulated health care professionals who promote health, well-being and quality of life by enabling individuals, families, organizations and communities to participate in occupations that give meaning and purpose to their lives” (Canadian Institutes for Health Information, 2022a). Physiotherapists or physical therapists (PTs), providing physiotherapy (PT), are “regulated, evidence-based, primary health care professionals who aim to prevent, assess and treat the impact of injury, disease and/or disorders in movement and function” (Canadian Institutes for Health Information, 2022b). Speech-language pathologists (SLPs), also referred to as speech therapists and providing speech language pathology (SLP), “work to prevent, assess, diagnose, and treat speech, language, social communication, cognitive-communication, and swallowing disorders in children and adults” (American Speech-Language-Hearing Association, n.d.). Physiatry, or physical medicine and rehabilitation, “is a medical specialty that emphasizes the prevention, diagnosis, treatment and rehabilitation of people disabled by disease, disorder or injury” (Association of Academic Physiatrists, n.d.). Due to the physical nature of their work, rehabilitation professionals are ideally positioned to identify signs of IPV that might otherwise be overlooked, and the trust they can develop with their clients over time gives them an opportunity to create a safe space for disclosure (Ballan & Freyer, 2019; Dalton, 2005; Stancliff, 1997). As such, it is imperative that rehabilitation professionals are equipped with the knowledge to identify IPV survivors and support them appropriately.

Among the physical injuries common among IPV survivors, brain injury is both highly prevalent and often overlooked (Campbell et al., 2022; Haag et al., 2022). Within the context of IPV, the substantial physical, cognitive, and mental health impacts of brain injury are exacerbated and can lead to longer-term disability if left unaddressed (Iverson & Pogoda, 2015; Iverson et al., 2017; Ponsford et al., 2014; Roberts & Kim, 2005; St Ivany et al., 2018; Valera & Berenbaum, 2003). Targeted rehabilitation intervention is critical for individuals with brain injury to achieve optimal outcomes and community integration (Sveen et al., 2022), further supporting the role of rehabilitation professionals with IPV survivors.

To our knowledge, no systematic investigation of rehabilitation for survivors of IPV exists in the published literature. To address this gap, this review explores four rehabilitation professions’ ((physiotherapy [PT], speech language pathology [SLP], and physiatry) knowledge and awareness of and interventions for IPV. Based on the literature identified, this review presents findings on three distinct categories of articles: (1) articles describing or evaluating rehabilitation interventions for IPV survivors; (2) articles assessing rehabilitation professionals’ knowledge or awareness of IPV; and (3) articles outlining opportunities for rehabilitation professionals to support IPV survivors. Given the high rate of brain injury among IPV survivors, the high rates of IPV among individuals living with disabilities including brain injury, and the usefulness of rehabilitation in this context, this review additionally reports on if and when brain injury is considered among articles across the three categories.

## Methods

This scoping review was guided by Arksey and O’Malley’s 2005 framework, which was expanded by JBI (formerly the Joanna Briggs Institute) (Peters et al., 2015; Peters et al., 2020) and the Preferred Reporting Items for Systematic Review and Meta-Analysis extension for scoping reviews (Tricco et al., 2018). As recommended by the JBI guidelines, a protocol is available via FigShare, an open science platform (Toccalino, Haag, Babineau, et al., 2022).

### Search Strategy

In all, 10 databases were identified in collaboration with an information specialist (JB) and searched for relevant articles using a search strategy including text words and subject headings (e.g., MeSH, Emtree) related to IPV and rehabilitation (see Appendix I). Search terms were informed by previous reviews exploring IPV (Toccalino et al., 2023) or the role of rehabilitation professionals (Chan, Estrella, Baddeliyanage, et al., 2022; Chan, Estrella, Syed, et al., 2022). The search strategy was first developed in Medline (see Appendix I) and subsequently translated to the databases. Database searches were last conducted in May 2022 and were not limited by language, year of publication, or geographic location. A manual search of the reference lists of included studies and scoping or systematic review articles identified through the search was conducted to identify additional relevant literature. A gray literature search of relevant IPV, gender-based violence, and rehabilitation organizations was also conducted to identify reports, policy briefs, clinical guidelines, or other unpublished work relevant to rehabilitation for survivors of IPV. The intention of the gray literature search was to capture existing practices being offered on an ad-hoc basis or under unique circumstances that might not be captured in the overall literature.

### Eligibility Criteria

Quantitative, qualitative, or mixed-methods research articles or dissertations/theses reporting primary data; commentaries; policy briefs; and clinical guidelines were included in the review if they met the following criteria:

1. Describe or evaluate:
  - an intervention provided by selected rehabilitation professionals (PTs, OTs, SLPs, and/or physiatrists
  - an opportunity for intervention by selected rehabilitation professionals OR
  - an educational or awareness intervention for selected rehabilitation professionals related to survivors of IPV AND
2. Focus on interventions for or about adult survivors of IPV (including domestic violence, dating violence, sex work, and sex trafficking).

Articles focusing on perpetrators of IPV, children, or youth under the age of 18, violence outside of an intimate partner relationship, or interventions provided by professions other than the four of interest (PT, OT, SLP, and physiatry) were excluded. Protocols, conference abstracts, book reviews, and studies conducted on animals were also excluded. Scoping and systematic reviews exploring relevant content were reviewed for relevant articles but were not included.

**Figure 1.**
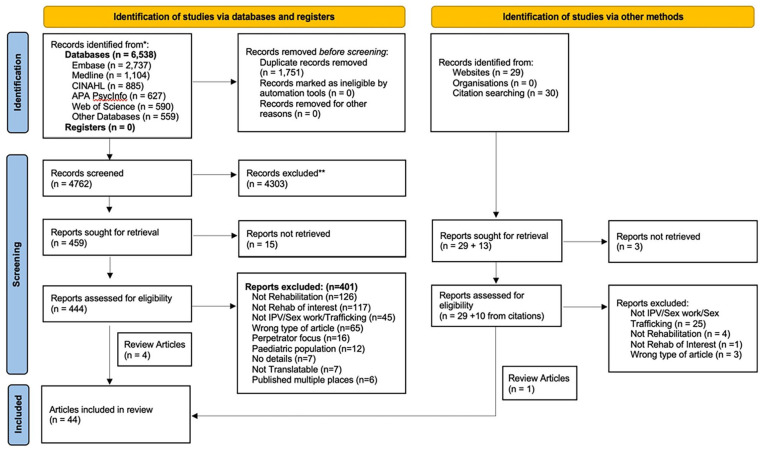
PRISMA flow diagram from Page et al. (2021).

Records were managed and duplicates were identified and removed using EndNote referencing software. Covidence Systematic Review Software was used to conduct the screening and monitor agreement between the reviewers’ assessments. Two reviewers independently screened titles and abstracts of all records and the full texts of potentially relevant articles based on the eligibility criteria outlined above. A small subset of articles was reviewed prior to commencing screening at each stage to ensure consistent application of inclusion criteria across reviewers. Differences in screening were resolved through discussion and consensus, or in consultation with a third reviewer if needed. Full texts of articles published in a language other than English were translated using Google Translate or DeepL Translate and the translated texts were reviewed. Articles in languages other than English that could not be translated (e.g., scanned text PDFs) were excluded. The PRISMA flow chart of the study selection process is presented in Figure 1.

### Data Charting and Synthesis

Article characteristics (e.g., year, country, type of article), study characteristics (e.g., setting, population, sample size, participant demographics), and information about the intervention or opportunity (e.g., target population, means of assessment, intervention details, outcomes) were extracted from included studies, with extraction completed by one reviewer and peer reviewed by a second. In addition, we noted if studies considered brain injury, including what assessment tools were used and what proportion of the study population had diagnosed or suspected brain injury. Descriptive statistics were used to convey article characteristics, and narrative synthesis (Popay et al., 2006) was used to synthesize study findings.

## Results

The database searches returned 6,538 records, from which the titles and abstracts for 4,762 unique records were reviewed, and 444 full texts were identified for further screening. An additional 29 records were identified through the gray literature search and 30 articles were identified from reference lists. A total of 44 articles met the inclusion criteria and were included in the review (see Figure 1 for a full breakdown). Articles were published between 1996 and 2022, predominantly in peer-reviewed journals (70%, *n* = 31) and based on data from the United States (86%, *n* = 38). Approximately half of the articles were primary research articles (48%, *n* = 21), with the remainder including clinical newsletters (20%, *n* = 9), commentary or overview-type articles (20%, *n* = 9), and statements by professional bodies (11%, *n* = 5), one of which pertained to a legislative bill.

**Figure 2.**
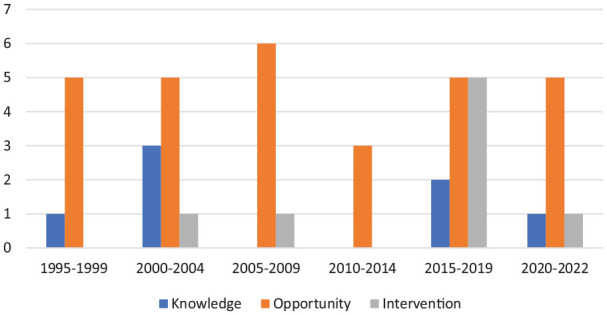
Distribution of articles by year and type.

Most included articles discussed opportunities for rehabilitation interventions for survivors of IPV (68%, *n* = 30). Eight articles (18%) described or evaluated rehabilitation interventions (Cerny et al., 2019; de Oliveira & Ferigato, 2019; Fitzgerald et al., 2017; Gutman et al., 2004; Helfrich & Rivera, 2006; Jones et al., 2021; Mangum et al., 2019; Walton et al., 2017), and seven articles (16%) explored rehabilitation professionals’ knowledge of or attitudes toward IPV (Clark et al., 1996; de Oliveira & Ferigato, 2019; Johnston et al., 2001; Macpherson et al., 2022; Shahgangar, 2004; Sivagurunathan et al., 2019; Williamson et al., 2004). One article touched on both rehabilitation interventions and knowledge and attitudes and is included in both categories (de Oliveira & Ferigato, 2019). Publication of articles discussing opportunities for intervention has been relatively consistent over time, while knowledge articles were more prevalent in the early 2000s and intervention articles have become more prevalent in the last 10 years (Figure 2). Most articles focused on IPV survivors (89%, *n* = 39), with a small minority focusing on sex trafficking (11%, *n* = 5 (Cerny, 2016; Cerny et al., 2019; Gorman & Hatkevich, 2016; Mangum et al., 2019; Thompson et al., 2020)). Most of the literature, and all articles focusing on sex trafficking, focused on the role of OTs in supporting survivors. For a summary of article characteristics and participant demographics, see Table 1. None of the included articles explored experiences of gender-diverse or two-spirit individuals, nor did the minority of studies reporting on the race or ethnicity of participants stratify findings or report on implications based on these aspects of identity. In the following sections, findings for each of the three article categories (rehabilitation interventions, knowledge and awareness of rehabilitation professionals, and opportunities for intervention) will be presented, followed by reporting on the recognition of brain injury in this body of literature.

**Table 1.** Summary of Article Characteristics.

	Knowledge, Attitudes, and Beliefs*		Opportunities		Interventions*		Total	
Article Characteristics	*N*	% of Category	*N*	% of Category	*N*	% of Category	*N*	% of Total
Number of articles	7		30		8		44	100
Primary research	7	100	7	23	8	100	22	50
Other	0	0	23	77	0	0	23	52
Rehabilitation profession								
Physiotherapy	2	29	6	20	1	13	9	20
Occupational therapy	3	43	20	67	7	88	30	68
Speech-language pathology	0	0	3	10	0	0	3	7
Physiatry	1	14	0	0	0	0	1	2
Combination	1	14	1	3	0	0	2	5
Survivor population								
Intimate partner violence	7	100	27	90	6	75	40	91
Trafficking	0	0	3	10	2	25	5	11
Country								
United States	4	57	29	97	5	63	38	86
United Kingdom§	0	0	1	3	2	25	3	7
Other†	3	43	0	0	1	13	4	9
Main participant group	Rehabilitation professionals		Survivors		Survivors			
Sample size								
Range	4–976		5–165,030		4–26			
Median	189		25		8			
Age of participants (range)	19–54+		18–60		18–64			
Sex of participants (%female)		24–90		94–100		100		
Reporting on race/ethnicity								
Yes	3	43	3	10	3	38	9	20
No	4	57	4	13	5	63	13	30
Not applicable	—		23	77	—			
Race/ethnicity of participants (among studies reporting)								
White/non-Hispanic		75–87		5–20		35–75		
Black		1		35–56		25–42		
Hispanic		5		4–24		15		
Other		16		—		8–25		

* de Oliveira and Ferigato (2019) included in both knowledge, attitudes, and beliefs and interventions categories.

§ Includes studies conducted in England, Wales, and the United Kingdom more broadly.

† One study conducted in each of Brazil, Canada, and Spain.

### Rehabilitation Interventions and Outcomes

Eight included studies focused on rehabilitation interventions, predominantly (*n* = 7, 88%) assessing OT interventions in support settings for survivors of IPV (*n* = 5) or human trafficking (*n* = 2). There was a high degree of variability across interventions, consisting of individual and/or group sessions that occurred in frequencies ranging from twice weekly to twice monthly with interventions lasting from several weeks to several months, and sample sizes were relatively low, ranging from 4 to 26. Most of the OT interventions assessed (*n* = 4, 63%) included individual sessions with intervention objectives highly tailored to the individual needs of participants (Cerny et al., 2019; Gutman et al., 2004; Helfrich & Rivera, 2006; Jones et al., 2021). These tailored interventions covered a variety of topics, but all focused at least in part on life skills such as goal setting, creating routines, communication, and stress or anxiety management. Two studies consisted only of group interventions, one focused on improving executive function (Mangum et al., 2019) and the other on psycho-education to improve self-esteem (Fitzgerald et al., 2017). All studies reporting on outcomes reported significant improvement in the targeted domain and/or high satisfaction among participating survivors; however, participant feedback from one of the shorter interventions suggested a 4-week intervention did not provide enough time to cover topics with the depth survivors wanted (Helfrich & Rivera, 2006). Across studies, there was significant variability in the assessments used and the objectives and delivery of the intervention. A total of 13 assessment tools were used across the nine studies, with the Canadian Occupational Performance Measure (Cerny et al., 2019; Jones et al., 2021; Mangum et al., 2019) and the Occupational Performance History Interview (Fitzgerald et al., 2017; Helfrich & Rivera, 2006) being the only two used more than once. Two additional articles did not discuss specific interventions. One was a broader qualitative exploration of the ways OTs assist survivors of domestic violence within primary care groups, noting the breadth of settings where OTs might support survivors (e.g., community workshops, home visits) and the variety of interventions OTs are equipped to provide (e.g., supporting self-expression related to violence, supporting social participation; de Oliveira & Ferigato, 2019). The final article described the evaluation of an IPV screening assessment for use in a PT clinic, noting strong inter-rater reliability and good construct validity for the tool (Walton et al., 2017). A full summary of rehabilitation intervention articles is shown in Table 2.

**Table 2.** Studies Examining Rehabilitation Interventions for Survivors of IPV and Sex Trafficking.

Article	Study Details	Assessment Tools	Intervention	Outcome
Cerny et al. (2019) USA *Peer-reviewed primary research*	Retrospective pre-/post-test design of **OT intervention** for women survivors (*N* = 15 started, *N* = 6 completed) in a transitional unit for survivors of **human trafficking** *Guiding Framework*: Model of Human Occupation	• Adolescent/Adult Sensory Profile • Sensory Modulation Screening Tool • Canadian Occupational Performance Measure	*Intervention details*: Individual sessions, 1×/week for 45–120 minutes, 12 weeks; some group sessions and informal interactions *Activities include*: meal preparation, creation of nightly routine, progressive muscle relaxation, promoting and modeling positive self-talk, daily planner creation and implementation, collage of life goals, parenting coaching and education, grounding activities, collaboration on resume creation, creation of individual sensory diets, leisure exploration, encouraging journaling and providing prompts, home modifications for calming, and self-expression	All clients reported an overall improvement in performance and satisfaction in their prioritized areas of occupation after 12 weeks
Jones et al. (2021) Wales *Gray literature program report*	Implementation and evaluation of **OT intervention** for female **IPV survivors** (*N* = 20) in a Domestic Abuse Safety Unit *Guiding framework*: Model of Human Occupation	• Occupational Circumstances Assessment Interview Rating Scale • Canadian Occupational Performance Measure	*Intervention details*: 2–20 individual sessions based on a tailored intervention plan (some interventions ongoing at the time of publication) *Topics included*: anxiety and/or depression management, sleep hygiene, relaxation techniques/visualization, mindfulness, goal setting, role development, assertive communication skills, developing new habits and routines, social interaction with family & friends, motivational interviewing, and social prescribing	15 people made significant improvements in their ability to perform their chosen occupations 18 people had significantly improved satisfaction with their ability to perform chosen occupations
de Oliveira and Ferigato* (2019) Brazil *Peer-reviewed primary research*	Qualitative participatory intervention research exploring **OT (*N*** **=** **4)** practices and intervention technologies in a primary care network in the interior state of São Paulo for women victims of violence	Not reported	*Main ways of approaching women*: individual care in OT, group care, income generation workshops, family care, home visits/care, matrix support, and therapeutic follow-up *Practice included*: supporting communication about the experience of violence; identifying occupational roles in daily dynamics contributing to the maintenance of DV; constructing strategies to deal with DV; (d) increasing social participation to increase self-perception, self-sufficiency, and women’s autonomy (e.g., income generation, self-care, strengthening social support networks)	Not reported
Fitzgerald et al. (2017) England *Peer-reviewed primary research*	Pre- post-test of a student-led **OT intervention** for female **DV survivors** (*N* = 8) participating in a DV coffee hour at a community center in the north of England	• Occupational Performance History Interview • Rosenburg Self-Esteem Scale • Quality of Life Rating	*Intervention details*: 5-week psycho-education group. *Activities included*: new skills and coping strategies, including mindfulness and cognitive behavioral therapy techniques, were utilized to support coping. Topics covered included the fight or flight response, assertiveness, the links between thoughts, feelings, and behaviors, effective communication, and the use of self-rating to help manage stress and anger	Self-esteem increased by 19% on average, and every participant’s score increased Participants demonstrated elevated occupational performance, self-esteem, and quality of life. All participants self-reported an increase in knowledge and that they would use the strategies learned in the future
Gutman et al. (2004) USA *Peer-reviewed primary research*	Quasi-experimental research design of an **OT intervention** for women (*N* = 26) with various disabilities who were homeless (*n* = 10) and/or experience(d) **DV** (*n* = 16) in a nonprofit facility in a large northeastern metropolitan area	• Individualized goal attainment scales created for each participant by the OT supervisors	*Group and individual sessions to address*: (a) Safety planning; (b) drug and alcohol awareness; (c) safe sex practices; (d) assertiveness and advocacy skill training; (e) anger management; (f) stress management; (g) boundary establishment and limit setting; (h) vocational and educational skill training; (i) money management; (j) housing application; (k) leisure exploration; and (l) hygiene, medication routine, and nutrition	High satisfaction was reported by 99%. Qualities ranked most important: client-centered and specific to each woman’s personal goals: intervention helped them take “baby steps” to learn new skills. Most influential aspects were anger management and stress reduction. 87.5% (14/16) achieved greater than their expected outcome (T scores > 50)
Helfrich and Rivera (2006) USA *Peer-reviewed primary research*	Clinical study of an **OT intervention** for women **IPV survivors** (*N* = 11) residing in an emergency homeless shelter in an urban Midwestern region *Guiding framework*: Model of Human Occupation	• Occupational Performance History Interview • Occupational Self-Assessment • Assessment of Motor and Process Skills • Assessment of Communication and Interaction Skills	*Intervention details*: life skills program delivered in group (1×/week) and individual (1×/week) sessions over 4 weeks *Activities included*: employment search support (interests and decision-making, job searching, mock interviews & prep, application prep, cover letter, and resume writing), job maintenance/advancement, FICA and taxes, minimum wage and the law, child labor laws, how to be a good employee, changing jobs: How and why?, Temporary Assistance for Needy Families, Victim’s Economic Security & Safety Act	Participants noted benefits included: being an outlet for discussion, to share frustrations, and receiving feedback and suggestions from peers. Shelter staff also provided positive feedback Area for improvement: more time is needed to sufficiently address the topics Overall, participant and staff feedback suggested that the clinical intervention was effective in providing participants with an opportunity to develop and enhance skills critical to obtaining and maintaining employment
Mangum et al. (2019) USA *Peer-reviewed primary research*	Pilot study with the pre–post-test design of an **OT intervention** for female residents (*N* = 8) of a local, 2-year residential program for survivors of **sex trafficking** in New Orleans	• Canadian Occupational Performance Measure • Executive Functional Performance Test • Occupational Therapy Task Observation Scale	*Intervention details*: 1-hour group sessions 2× per month *Activities included*: specific tasks performed to completion that emphasized skills such as problem-solving, decision-making, frustration tolerance, appropriate pacing, attention, inquiring, sequencing, gathering, organizing, adjusting, responding, enduring, initiating, and cooperating	Four participants completed post-tests. 75% (3 of 4) reached minimally clinically important differences in occupational performance—notable improvement in executive function. Significant gains were noted in several subtest areas of the OT task: frustration tolerance, problem-solving, following directions, expression, and cooperation
Walton et al. (2017) USA *Peer-reviewed primary research*	Study to determine validity and reliability of **IPV** screening survey for use in **PT clinic**	Survey screening tool	N/A	Strong inter-rater reliability between experts, strong internal consistency, and percent agreement; suggests good to excellent construct validity for this screening tool

*Note*. OT = occupational therapy; PT = physiotherapy; IPV = intimate partner violence; DV = domestic violence.

* Reports on both interventions and knowledge and attitudes, and is therefore represented in both tables. Bold text indicates profession (e.g., OT) and survivor group (e.g., IPV) of interest.

### Assessment of Knowledge and Attitudes of Providers

Seven of the included studies explored knowledge and attitudes about IPV among rehabilitation professionals, with studies predominantly focused on PTs (*n* = 2), OTs (*n* = 3), or a combination of the two (*n* = 1); however, the only study included in this review focusing on physiatrists/physical medicine and rehabilitation physicians was included in this category (Williamson et al., 2004). Most studies (*n* = 5, 71%) assessed knowledge or attitudes using questionnaires that were adapted from previous studies (Johnston et al., 2001; Shahgangar, 2004; Sivagurunathan et al., 2019; Williamson et al., 2004) and/or developed based on literature reviews (Clark et al., 1996; Johnston et al., 2001). There was minimal overlap in the questionnaires used, though two studies used Tilden et al. (1994) survey on experiences and attitudes toward family violence (Johnston et al., 2001; Williamson et al., 2004). The two remaining studies were a case analysis (Macpherson et al., 2022) and a qualitative exploration using semi-structured interviews (de Oliveira & Ferigato, 2019). Overall, rehabilitation professionals reported being under-prepared to deal with IPV (52-68% of respondents (Johnston et al., 2001; Williamson et al., 2004)) with little to no formal education on the subject (65%–100% of respondents (de Oliveira & Ferigato, 2019; Johnston et al., 2001)). Between 34% and 76% of professionals reported they had not, to their knowledge, encountered IPV survivors or had little experience supporting them within their practice (Johnston et al., 2001; Shahgangar, 2004; Sivagurunathan et al., 2019). When asked, the majority of rehabilitation professionals wanted more information or training about IPV and ways to support survivors (Clark et al., 1996; Shahgangar, 2004), including more knowledge of local laws and services (Williamson et al., 2004). Service providers also preferred practical and team-based approaches to supporting survivors (de Oliveira & Ferigato, 2019; Macpherson et al., 2022). Recommendations across studies focused on the need for more education to be available both through curricula and continuing education opportunities. There was also a noted need for protocols or procedures to help rehabilitation professionals identify IPV among their client base and evaluations of the efficacy of these protocols and the educational programs. A full summary of knowledge and attitudes articles is shown in Table 3.

**Table 3.** Studies Examining Knowledge of and Attitudes Toward IPV Among Rehabilitation Professionals.

Article Details	Study Details	Assessment Tools	Key Findings	Next Steps
Clark et al. (1996) USA *Peer-reviewed primary research*	Survey (1994) to describe **PTs’** (*N* = 151: female *n* = 121, 80%; male *n* = 30, 20%) knowledge and recognition of **battered women.** Academic degrees (from certificates in PT to master’s degree); 0.3–24 years of experience	13-item questionnaire based on a literature review, divided into three sections: demographic information, knowledge of battering symptoms, and past recognition of battered patients	• 43% treated female patients suspected or confirmed as being battered; the majority of these (52%) gave the patients information regarding shelters or counseling. • 76% identified books or magazines as their source of DV information; only 8% reported receiving training in school • 42% correctly identified the head, neck, chest, and abdomen as the most common locations of DV-related injuries • 58% never asked patients if they were physically battered (1% asked routinely) • 86% (*N* = 146) stated they would benefit from more instruction regarding DV identification and intervention	PTs rarely suspect DV in their patients or report to authorities; this can be due to a lack of inquiry or lack of instruction on DV reporting in schooling *Solution*: Develop and assess the efficacy of DV detection protocols and educational programs for PTs More research surveying PT schools to determine whether education on DV is being included in PT curricula
de Oliveira and Ferigato* (2019) Brazil *Peer-reviewed primary research*	Qualitative study to identify and analyze 6 **OT’s** (*N* = 4 from 30 to 54 years) practices and intervention technologies in **women victims of violence** in Sao Paulo. 3 attended public universities and 1 has no postgraduate degree	Semi-structured interviews lasting ~60 minutes were audio-recorded, transcribed, and coded	All interviewees said they did not adequately discuss violence against women during graduate or other training. This was reflected in technical, personal, and social difficulties perceived by OTs with a lack of a practical approach to the problems of violence against women	Necessary for OTs to have ethical and political training beyond the technical dimension of the work process, bringing part of the responsibility for social transformation and incorporating the practice of making notifications in the daily routine of health service. *Solution*: Explicitly incorporate comprehensive care and gender issues in OT training, increase awareness of women’s care network mechanisms
Johnston et al. (2001) USA *Peer-reviewed primary research*	Cross-sectional survey to determine if **OTs** (*N* = 202: female *n* = 187, 93%; male *n* = 15, 7%) possess the ability to identify **wife abuse** by measuring their knowledge and attitudes about such abuse. *87% White, non-Hispanic.* **OT:** 78% **COTA**: 22% experience: 11 years (avg.) BA earned 57%.	69-item, closed-ended format questionnaire based on literature review and previous work, divided into three sections: demographic information including personal experiences with abuse, knowledge of wife abuse, and attitudes/beliefs about abuse measured with Likert scale	• 65% reported no formal and 76% reported no clinical education about wife abuse. • 68% did not feel adequately prepared to identify cases of wife abuse, and 70% did not feel prepared to provide either referrals or interventions to women being abused. • Significant positive correlations between average knowledge scores and attitudes of wife abuse; between age and attitudes of the role of OT in identifying wife abuse. • Female OTs had significantly more empathic attitude toward wife abuse	*Solution*: Academic programs should expand curricula to include wife abuse, increased continuing education on the topic. Identification of wife abuse is key, OTs should commit to treating the whole person and individualizing treatment. To do so, it is important that practitioners use interactive reasoning when collaborating with their patients
Macpherson et al. (2022) Spain *Peer-reviewed primary research*	Mixed qualitative–quantitative exploratory study to explore the ethical dilemmas that 298 Spanish undergraduates (**PT**: *n* = 109: female *n* = 51, male *n* = 58; nursing, dentistry) students aged 19–21 (2nd/3rd year) endure when supporting survivors of **IPV** between October 2014-April 2016.	CBL: clinical case described a female patient with partner-inflicted injuries to the mouth and the hip. When healthcare staff urges her to inform the authorities, she outwardly refuses, saying that it is none of their business, that she knows how to deal with her partner and that she would deny everything, so they stop questioning her. [Individual evaluations ~30 minutes]	• 44% of PTs would have a personal conversation and psychologists/social workers’ assistance; significantly more than dentistry students (7%) • 8% would only use a personal conversation; significantly fewer than both nursing (30%) and dentistry (53%). Students with lower levels of ethics prefer personal conversation only and a higher level of ethics training favor personal conversation combined with psychological or social assistance. No significant differences were found between responses provided by men and women	~70% lean toward personal conversation, generally supported by other measures. This result suggests that healthcare students value the capacity for dialogue. It may also manifest the lesser choice of the students to involve the legal authorities, psychologists, or social workers without engaging in an initial conversation with the patient. This is particularly important given the fact that these healthcare students may not necessarily be proficient in conversational methods with patients
Shahgangar (2004) USA *Thesis*	Quantitative survey to determine the level of knowledge and attitudes of **OTs** (*N* = 81: female 90%; male 10%) regarding **DV and battered women** in the Dallas-Fort Worth (DFW) area. **OTR** (88%) and **COTA** (12%) presently residing in the Dallas-Fort Worth area. Between 31 and 50 years of age	37-item modified version of existing Questionnaire on DV	• 60% reported coming across <1 case of DV per year in their practice • 58% had never attended any lectures or seminars about DV; only 43% of the knowledge questions were answered correctly • Majority identified it is a crime to hit your partner (60%) or force sex on your partner (63%), that DV occurs in homosexual relations (79%) • 19% of respondents had information pamphlets or used a written protocol for DV cases • 38% did not know whether DV was more common in ethnic minorities • 94% agreed DV is an important healthcare issue and 59% agreed OTs should be more involved in identifying DV • 71% would welcome training on DV • Participants had a positive attitude but lacked skills in discussing DV with patients	OT have general knowledge of DV but lack specific knowledge & training. 50% of OT reported feeling uncomfortable discussing DV with patients. Respondents who were more aware of DV had supportive and positive attitudes regarding DV. Research suggests that OT should have basic knowledge and understanding of DV to improve the quality of care and interventions provided to survivors of DV
Sivagurunathan et al. (2019) CAD *Peer-reviewed primary research*	Cross-sectional online survey to describe the attitudes and beliefs of 189 **hand therapists (HTs**, *N* = 189, 86% **OT**, remainder **PTs**) about **IPV** between February and April 2015. Female: 89.2% Male: 10.8%. Mean age = 49, range (24–73) Years in practice (23.7) and years in certified hand therapy (17.6)	27-item questionnaire that was modified from the two surveys previously published by Maiuro et al., (2000) and Connor et al., (2011). While minor modifications in wording were made to focus on HTs, essential constructs of all items were retained	• 66% had prior experience with IPV • HTs reported neutral perceptions about self-efficacy, client or personal safety, and support systems available when addressing IPV in practice. However, therapists considered intervening as part of their professional role and reported low levels of victim-blaming attitudes. Those with firsthand IPV experience reported lower victim blaming. • Women and HTs with firsthand experience are significantly less likely to blame victims of IPV (only comparisons with significant differences)	HTs believe their role includes addressing IPV, confidence to deal with IPV, access/awareness of resources, and perceived safety were barriers to screening for IPV *Solution*: Research should identify effective tools to educate and assist HTs to identify and support survivors of IPV Target male HTs with educational strategies aimed at reducing victim blaming. Involve individuals with firsthand experience of IPV in knowledge translation efforts
Williamson et al. (2004) USA *Peer-reviewed primary research*	Qualitative survey to determine attitudes and behaviors of physicians (*N* = 976; **physical medicine/rehabilitation** *n* = 34) in Arizona about **DV** by looking at screening practices and motivating factors and barriers to providing **DV** screening and to design educational interventions Female: 233 (23.9%); male: 738 (75.6%). Mage = 48.2 *African American: 13 (1.3%); American Indian: 10 (1%); Asian/Pacific Islander: 91 (9.3%); Hispanic: 49 (5%); White/non-Hispanic: 762 (78.1%).* Years in practice ranged from 1 to 57 (*M* = 16.38)	23-item questionnaire based on previous studies Designed to be completed in 5 minutes or less. Most questions were multiple-choice on Likert scales. A few open-ended questions were also asked	• Physical medicine/rehabilitation among six specialties scoring lowest on DV education, screening, awareness of services, and competence in treating • Physicians broadly reported that they were willing to use DV materials in their practice (82.6%) and were comfortable using a screening tool (76.5%). • 86.8% indicated that DV is primarily a social problem (vs. medical [12.3%] or legal [7.4%]), 49.9% did not identify any barriers to screening. 48% were not aware of or knew little about services for survivors. 52% of the respondents in our study rated their competence at providing care as poor to fair • 67.7% would prefer presentations by advocacy groups, and 41.9% wished to hear about laws regarding DV. Physicians were less interested in statistics on DV homicides/suicides (29.6%) and personal stories from survivors (20.6%)	There is a need to provide quality DV education and practical information to both primary and specialty physicians and points to some methods of customizing this training

*Note*. CBL = case-based learning; COTA = certified occupational therapist assistant; DV = domestic violence; IPV = intimate partner violence; OT = occupational therapy; OTR = registered occupational therapists; PT = physiotherapy.

* Reports on both interventions and knowledge/attitudes/beliefs, and is therefore represented in both tables. Bold text indicates profession (e.g., OT) and survivor group (e.g., IPV) of interest.

### Opportunities for Rehabilitation Interventions

Included articles identified opportunities for rehabilitation professionals to support survivors that broadly grouped into three themes: identification or screening, referral, and support. Identification or screening was discussed in nine of the included articles (Ballan & Freyer, 2021; Dalton, 2005; Foose, 1999; Gaffigan-Bender & Narula, 1998; Gallew, 2004; Helfrich et al., 2001; Johnson, 1997; No Author (APTA Guidelines), 1998; Stancliff, 1997), noting rehabilitation professionals as being in an ideal position to identify survivors because of the nature of the physical assessments they conduct (Ballan & Freyer, 2019; Dalton, 2005; Helfrich et al., 2001; Stancliff, 1997) or the relationship that can be developed between practitioner and client over time (Ballan & Freyer, 2021; Dalton, 2005; Helfrich et al., 2001; Johnson, 1997; Stancliff, 1997). Some authors advocated for embedding screening into routine practice (e.g., Dalton, 2005). Several studies further recommended that individual practitioners or the institutions in which they work should develop and maintain lists of local resources to which they could refer clients when needed (Gallew, 2004; Helfrich et al., 2001; Javaherian, 2006; Javaherian et al., 2007; Javaherian-Dysinger & Underwood, 2011; Javaherian-Dysinger et al., 2017; Johnson, 1997; No Author (APTA guidelines), 1998). In this way, rehabilitation professionals can be prepared to identify and support the survivors who are invariably among their clientele.

Beyond identification and referral, articles discussed many ways in which rehabilitation professionals can support survivors, ranging from relatively small changes within a practice such as having pamphlets on domestic violence available in washrooms or waiting rooms (Helfrich & Aviles, 2001; Javaherian, 2006; Johnson, 1997), to broader institutional changes such as developing procedures or policies regarding IPV or sex trafficking (Ballan & Freyer, 2021; Dalton, 2005; Javaherian, 2006; No Author (APTA Guidelines), 1998). Recommendations for rehabilitation professionals providing care include approaching the conversation in a nonjudgmental way (Gaffigan-Bender & Narula, 1998; Gallew, 2004; Johnson, 1997; Kessler, 2012; Stancliff, 1997), assessing for safety (Helfrich & Aviles, 2001; Koch, 2001; No Author (APTA Guidelines), 1998), and using a trauma-informed approach (Ballan & Freyer, 2019, 2021; Cerny, 2016). At both the individual practitioner level and the institutional level, a collaborative approach to care including rehabilitation professionals, mental healthcare providers, community organizations, and other healthcare providers was encouraged to better support survivors (Ballan & Freyer, 2019, 2021; Foose, 1999; Kessler, 2012). Finally, more education for rehabilitation professionals during their degrees and opportunities for ongoing professional development and training postgraduation were also deemed critical by many authors (Ballan & Freyer, 2021; Cerny, 2016; Dalton, 2005; Gallew, 2004; Javaherian, 2006; Johnson, 1997; No Author (APTA Guidelines), 1998). A full summary of primary research articles and perspective or recommendation articles discussing opportunities for rehabilitation professionals to support survivors is found in Tables 4a and 4b, respectively.

**Table 4a.** Studies Discussing Opportunities for Rehabilitation Professionals to Support Survivors of IPV and Sex Trafficking (Primary Research Studies).

Article Details	Study Details	Assessment Tools	Intervention/Interventionist Details	Outcome
Ballan and Freyer (2020) USA *Peer-reviewed primary research*	Qualitative study to examine the impact of occupational deprivation on 25 female survivors of **IPV** who have physical disabilities in NYC (implication for **OT**) Age: 18–60 Asian-American: 8% (*n* = 2) African-American: 44% (*n* = 11) Hispanic/Latino: 20% (*n* = 5) White, non-Hispanic: 20% (*n* = 5) Other: 8% (*n* = 2)	Semi-structured interviews • abuse experience and risk assessment • use and non-use of self-protective strategies • necessary support services • impact of disability	OTs should familiarize themselves with the signs and symptoms of IPV among survivors with disabilities to heighten awareness of its occurrence among their clients. OTs can assist IPV survivors with disabilities and DV agencies to heighten occupational engagement and independent functioning	Occupational deprivation used against women by their partners to isolate them Occupational deprivation compounds the negative impacts of IPV, particularly among women with disabilities. Working with survivors with disabilities to optimize occupational functioning and enhance independence may be among the most important interventions to provide women with tools to leave abusive situations
Ballan et al. (2021) USA *Peer-reviewed primary research*	Retrospective cross-sectional study to facilitate a better understanding of the relationship between **IPV** and occupational functioning among 205 survivors (93.7% female) with disabilities who were current or former **DV** shelter residents and opted for an **OT** assessment between January 2013 and December 2018 in the US (implication for **OT**) Age: 19–59. White: 4.9% (*n* = 10) Black: 47.3% (*n* = 97) Latinx: 34.6% (*n* = 71) Asian: 3.9% (*n* = 8) Other: 2.9% (*n* = 6)	All measures except for the HELPS screening tool (Picard et al., 1991) were developed by the study site for internal use and do not have established psychometric properties	Positive TBI screen had higher mean emotional/cognitive functioning scores (higher score = greater difficulty). 24.5% (*n* = 51) screened positive for a possible TBI (HELPS)—these participants were more likely to report difficulty participating in life roles and poorer emotional/cognitive functioning than those who did not Significant relationships were found between measures of functioning and participant abuse experiences and disability diagnoses, and descriptive results regarding unemployment, abusive relationship duration, and type of abuse Significant relationships were found between participation in life roles and the type of abuse experienced, as well as ADL execution, emotional/cognitive functioning, and difficulty participating in life roles. Disability type, a positive TBI screen, and resident abilities were likewise significantly associated with emotional/cognitive functioning scores	OT interns assisted survivors with goals in six distinct areas: work/volunteer/education skills, cognitive skills, life skills, improving physical function, leisure exploration/social skills, and referrals to additional programs if needed While the majority of study participants were able to execute ADL independently, participants reported more need for assistance with IADL including money management, work, time management, and leisure activities. Furthermore, the majority of the sample was unemployed. Residents who screened positive for possible TBI were more likely to report difficulty participating in life roles and poorer emotional/cognitive functioning than those who did not; Agencies working with IPV survivors are often trained to recognize mental health issues but are not familiar with signs of TBI, and thus may fail to address the damaging effect of brain injury
Humbert et al. (2014) USA *Peer-reviewed primary research*	Phenomenological study to explore how women experiencing **IPV** anticipate their recovery process, and to explore 8 women’s perceptions of the challenges that influence that process (implication for **OT**) Age: 20–48	In-depth semi-structured interviews utilizing the Kawa Model to support survivors of **DV** (Iwama, 2006)	OTs working with women recovering from IPV may be qualified to intervene with these issues to facilitate a less-obstructed path to recovery Five themes were primarily set within the participants’ illustrations. These themes were: I Want to Make a Better Life for My Children, This Will Make Me Stronger, I Have to Try to Get Stability in My Life, Learning How to Have a Relationship with Myself, and I Know That I Can Do It on My Own. The remaining theme is, I Know in My Heart That It Gets Better. Despite the difficulties, many of the women expressed a general belief that their lives would improve drastically in the future	Main obstructions to their recovery were pragmatic in nature. Some obstructions and challenges included education, finances, homelessness, and employment. Researchers were unable to explore OT implications specifically used with this population during the study. The nature of the obstructions and challenges reported by participants indicate that it may be an important area for further research. The themes that emerged in the findings alluded to the participants’ desires to lead independent lives in which their needs and the needs of their children could be met without interference
Javaherian et al. (2007) USA *Peer-reviewed primary research*	Phenomenological research to contribute to **OTs’** understanding of the experience of **DV**, its impact on the lives of 5 women, and the needs of these women as they journey toward independent and abuse-free lives in a Midwestern state. Age: 19–52	Questionnaire	Five themes emerged to describe the women’s experiences:(1) “You owe yourself a life,” (2) “It’s really all about connecting the dots,” (3) “I don’t have an ounce of time to myself,” (4) “It gets hard,” and (5) “That was the road I traveled but this is now the road that I’m on.” These themes emerged from the voices and stories of the women are quotes that reflect the women’s struggles to leave the abuser, enter the shelter, and begin the process of rebuilding their lives	OT practitioners can help survivors of DV build self-esteem, self-efficacy, and explore coping strategies OT programming can address parenting strategies and money and time management, leisure and job exploration, self-advocacy, and increasing self-esteem, accessing community resources, and promoting the parent–child relationship Women with children may take longer to cope and recover from abuse than those who do not have children
Javaherian-Dysinger et al. (2016) USA *Peer-reviewed primary research*	Retrospective study to describe the occupational needs and goals of 68 women residing in a **DV** shelter and their self-perceived changes in satisfaction and occupational performance in southern California between 2007 and 2013. (implications for **OT**) Age: 18–50	Semi-structured interview COPM interview-based Community-based Practice Evaluation	OT group interventions offered 2–3 times per day at least 4 days a week. Groups were occupation-based and emphasized active engagement targeting life skills, health, and self-esteem. All women also offered individual sessions 1–3 times per week to address personal goals. Comparison of pre–post self-rated performance and satisfaction in the OT therapy groups and individual sessions appeared to improve survivor's self-rated satisfaction and performance in their goal areas. Women benefited from hands-on groups Women frequently reported a need for organizational skills and legal assistance. Participants rated problems in the area of work, social participation, IADL, and education as most important on a scale of 1–10.	OT interventions coupled with other shelter classes on DV demonstrated significant changes in both satisfaction and performance scores. Groups and individual sessions are tailored to address the needs identified. Limitations inherent to the retrospective design restricted the data and its generalizability; future studies should further explore the effectiveness of OT interventions and treatment protocols
O’Neil-Pirozzi (2003) USA *Peer-reviewed primary research*	Qualitative study to examine sleep language abilities of 25 women and their children (17 boys and 12 girls) in homeless shelters where **DV** is among the most common cause of homelessness in Boston and Newport. (implication for **SLP**) Mothers’ age: 21–43 African American: 56% (*n* = 14) Hispanic: 24% (*n* = 6) Caucasian: 20% (*n* = 5)	Pure tone hearing test Diagnostic batter of auditory comprehension, oral expression, reading, and writing abilities	3–4 women had a medical history of head injury (BI considered) The communication impacted was oral expression and writing as well as expressive modalities	Women have areas of language deficiency that may benefit from SLP support. Research on the effectiveness of language-based intervention programs is needed. Language abilities of these mothers facilitated their academic, psychosocial, societal, and vocational success, which would also be expected to have a positive impact on their children
Smith & Strauser (2008) USA *Peer-reviewed primary research*	Secondary analysis of survey data to examine the relationship between the employment rate of 165,030 women in the USA with disabilities and the incidences of physical and sexual violence in the USA (implications for rehabilitation professionals broadly)	Behavioral Risk Factor Surveillance System questionnaire	Women with disabilities who have experienced IPV have significantly higher levels of unemployment. Education is a strong protective factor for women with disabilities who experienced IPV. Being a non-white woman with disabilities had an increasing likelihood of experiencing unemployment	Therapists need to be aware of signs of abuse and proper protocols. Abuse screening, intake, and evaluation are important. Psychological assessments should be completed. Collect outcome data on whether intervention was appropriate or not.

*Note*. COPM = Canadian Occupational Performance Measure; IADL = instrumental activities of daily living; OT = occupational therapy; PT = physiotherapy; OTR = registered occupational therapists; COTA = certified occupational therapist assistant; Mage = median age; DV = domestic violence; IPV = intimate partner violence.

**Table 4b.** Studies Discussing Opportunities for Rehabilitation Professionals to Support Survivors of IPV and Sex Trafficking (Perspective Articles).

Article Information	Role of Rehab Professional	Recommendations for Rehab Professionals	Recommendations for Educators/Agencies
Ballan and Freyer (2019) (Perspectives/Recommendations) *SLP, IPV* *Women with Disabilities* *Support*	• Close focus on the head, neck, and mouth enables SLPs an opportunity to identify physical injuries/signs of trauma from IPV • SLPs can assist with communicating disclosure through the development of speech, language, and communication skills; assisting with self-expression and enabling alternative forms of communication if verbal communication is a challenge	• Trauma-informed approach, including giving patients full control over how and in what manner they will be touched • Be aware that communication disorders can be an indicator of trauma or abuse • Assess for brain injury in survivors of IPV (HELPS screening tool) • Conduct cognitive-communication evaluation if needed (e.g., Cognitive Communication Checklist for Acquired Brain Injury) • Collaborate with the survivor on treatment plan and reporting • Collaborate with community agencies and other service providers with expertise to address the relevant needs of IPV survivors with communication disorders	
Ballan and Freyer (2021) (Perspectives/Recommendations) *PT, IPV* *Women with Disabilities* *Screening & Referral* *Support*	• IPV survivors are more likely to present with chronic conditions that might be addressed by PT • PTs have specialized skills to improve functioning, could be supportive to women with disabilities leaving abusive situations • Longer-term relationships with PTs than other health professionals (emergency room, physician)	• Screen for IPV in all patients and create safe space for disclosure: IPV Screening Tool for PTs; Abuse Assessment Screen—Disability • Use a trauma-informed approach (safety, trustworthiness, choice, collaboration, and empowerment) • Recognize interrelatedness of physical and psychological concerns, work toward a “positive connection between survivors and their bodies” • Collaborate with mental healthcare providers, community agencies, and healthcare settings to provide a holistic approach and ensure appropriate referral pathways are available	• Ensure adequate training and continuing education on recognizing and responding to abuse, particularly the increased risk among women with disabilities • Ensure agency policies about addressing IPV are explicit
Cerny (2016) USA (Perspectives/Recommendations) *OT, ST* *Support*	Support ADLs, IADLs, meaningful leisure, parenting, managing stress, gaining & maintaining safe employment & housing, etc. *Guiding Framework*: Person-Environment-Occupation Model	• OT interventions for adult survivors of DV or adults at risk for homelessness can be useful for survivors of trafficking • Be aware of trauma-informed, culturally appropriate care, and identification of victims • Holistic and survivor-focused interventions, provide client-centered care for current and changing needs • Empower survivors of human trafficking to address the intrinsic and extrinsic barriers to achieving increased overall well-being and occupational performance	• Educating OT students about human trafficking and the possible occupational injustices occurring as a result • Universal and targeted services taking place at the community level may include evaluating school and community settings for the presence and quality of efforts related to human trafficking awareness, prevention, and victim referral
Dalton (2005) USA (PT magazine article) *PT, IPV* *Screening*	• Screen for DV and provide proper resources • Provide a supportive and open environment to engage (if/when the patient is willing)	• Embed screening into routine practice • *“we are in a position where people see us over time, they trust us, and they may be likely to tell us things they won’t tell casual health care providers”* • Look for physical signs of abuse and other red flags (e.g., unusual bruising, history of multiple broken bones, non-adherence to treatment)	• Education and training on family violence including challenging assumptions (e.g., that leaving is easy, stereotypes of what a victim of abuse looks like) • Develop or implement facility-wide policies on DV and create environments supportive of collaborative teams to care for DV survivors • Meaningful connection with patients and the community around DV and other topics
Excerpts from APTA’s Guidelines (1998) USA (PT magazine insert) *PT, IPV* *Screening & Referral*	• Recognize and report DV, refer to appropriate community resources • Ensure survivors’ rights, dignity, and confidentiality are upheld *“The most helpful thing anyone can do is listen to them, believe them, and take them seriously”*	• Ongoing reassessment of safety and mental health needs and awareness of pharmacotherapy • Evaluation and examination should be conducted with the consent of the patient and in line with the facility’s policies for collection, retention, and safeguarding of evidentiary material • Be aware of signs and physical injuries that might be indicative of abuse (e.g., partner interference in medical care, noncompliance with treatment, repeated or chronic injuries, chronic pain)	• Facilities should develop objective criteria for identifying victims of DV; all individuals involved in screening should be knowledgeable on criteria for identifying and supporting DV survivors • Supervisors should be responsible for providing training and continuing education on identification and support of DV survivors • Develop and maintain: a list of community agencies for referrals; a DV protocol for emergencies
Foose (1999) USA (PT magazine article) *PT, Elder Abuse/IPV* *Screening*	PTs are in an ideal position to recognize abuse and who is inflicting it	• Elder abuse by a partner can be (inadvertent) neglect or deliberate abuse. With neglect, PT needs to be aware of the limitations of their partner and how that might impact their caregiving capabilities	• Facilitate an interdisciplinary approach, involving social work, nursing, physicians, PTs, OTs, and SLP.
Gaffigan-Bender and Narula (1998) USA (continuing education article) *PT, IPV* *Screening*	PTs should screen for DV and create a safe, understanding, and nonjudgmental space for disclosure	• Ask direct questions about injuries, evasive behavior, and patient’s fear of partner (disclose mandatory reporting laws prior to asking) • Confidentiality is essential—notes about DV should not go into insurance forms or discharge instructions • Have an understanding, nonjudgmental approach, emphasize nothing justifies abuse • Ask about safety, suicidality	
Gallew (2004) USA (OT Practice—magazine) *OT, IPV* *Screening & Referral* *Support*	• OTs must understand and identify signs of DV, to provide appropriate resources and referrals • OTs can use their expertise to promote performance and life participation	• Be aware of and able to refer to local resources • Create a safe environment, pose questions that allow for disclosure, and assure survivors there is no excuse for violence • Ask about resources and support system; continue regular support with survivors • Promote occupational justice (right to participate in meaningful and valued occupations)	• Further education is needed to support male victims of DV (the focus of the article is on women) • OT, as a profession, must reflect on its founding values and domain of practice to recognize its crucial role in working with survivors of DV to promote life participation and health
Gorman and Hatkevich (2016) USA (Perspectives/Recommendations) *OT, ST* *Support*	OTs have a role in rehabilitating victims and survivors of human trafficking and advocating for ST prevention	• OT services may include assessing and improving physical and psychological symptoms, improving performance in occupational areas, and developing healthy performance patterns using client-centered and occupation-based methods Javaherian-Dysinger et al. 2011. • OT interventions may include addressing musculoskeletal problems and providing training in compensatory techniques related to memory loss and other cognitive issues deriving from trauma, environmental deprivation, and lack of education	• Development and piloting of occupation-based programs and interventions for ST survivors is critical • Development of outcome assessments for OT services with ST survivors • OTs can play a role in ST prevention education, increasing awareness of ST, and advocacy
Helfrich and Aviles (2001) USA (Perspective/Recommendations) *OT, IPV* *Support*	OTs will work with DV survivors across settings in varying capacities depending on the needs of the client and the therapist’s skill and attitude—the variability in the latter needs to be removed *Guiding Framework*: Model of Human Occupation	• Assess for safety in the home and current living situation • Provide services for current needs, but also be aware of future needs (e.g., will needs change when they leave the abuser, the shelter) • Be aware of local resources for referrals • Indirect support via training DV agency staff **OT Tools** • The Occupational Performance History Interview • The Occupational Self-Assessment • The Assessment of Motor and Process Skills • The Assessment of Communication and Interaction Skills	• Have DV information in women’s restrooms within the OT clinic area so women can learn about DV without their abuser’s knowledge • Program consultation with DV agencies to develop programs or expand intake to include functional assessment
Helfrich et al. (2001) USA (Perspective/Recommendations) *OT, IPV* *Screening & Referral*	OTs are often in positions to see physical indicators because of the nature of their relationship with clients, must be able to respond appropriately	• Be aware of indicators of DV (inconsistency between injury and explanation, central pattern of injuries, bruised in various stages of healing, hiding injury, dismissive/defensive about injury) • Have resource and referral lists to provide to clients • Evaluate ability to perform, rather than basing their evaluation on the person’s report of their performance	
Javaherian-Dysinger et al. (2017), Javaherian-Dysinger and Underwood (2011), Javaherian et al. (2007) USA (AOTA Statements) *OT, IPV* *Referral & Support*	• OTs are responsible for the evaluation, intervention process, and interpretation of assessment results • OT practitioners can provide direct or indirect services to local DV centers through individual evaluations and interventions, life skills groups, advocacy, and consultations *Assessment tools*: • Occupational profile • Community-Based Occupational Therapy Evaluation • HELP or HELP–Screener • Adult Sensory Profile	• Filing a report to the local law enforcement agency or children’s or adult protective services • Interviewing, evaluating, and providing interventions without the abuser present to allow the client to discuss the situation in relative safety • Identifying and assessing injuries and potential causes • Talking to the client about healthy relationships and addressing areas of occupation, performance patterns, and skills that may have been affected by the abusive relationship, such as leisure, work, IADLs, and ADLs • Respecting the client’s perception of the relative danger of the situation to their life and the well-being of other family members, remaining empathetic and nonjudgmental about the client’s decision to remain in or leave the abusive situation • Providing the client with contact information for the local DV hotline • Following safety precautions to determine whether it is appropriate to conduct home visits	• Develop a collaborative plan (OTs and OT assistants) for supervision to guide evaluation and intervention processes • Advocacy training is essential. Practitioners are academically prepared to work with survivors in a variety of contexts given their knowledge of development, behavioral health, and health promotion
Javaherian (2006) USA (OT Practice—magazine) *OT, IPV* *Support*	• Support active and healthy participation in ADLs as well as IADLs • Explore independent client factors or performance and skills to address the impact of DV on their occupational ability and performance.	• Initiate conversations surrounding DV to provide an opportunity for them to recognize that the OT is available to support them • Be familiar with state laws around reporting suspected DV	• Joint Commission on Accreditation of Healthcare Organizations (JCAHO) requires employees to demonstrate education and training in DV • Have DV-related resources (DV organizations, shelters, etc.) in OT office spaces and washrooms in case the abuser attends appointments • DV involving men as victims and in same-sex partnerships may require special attention
Johnson (1997) USA (PT Magazine article) *PT, IPV* *Screening & Referral*	• PTs have the opportunity to identify and intervene with regard to DV • PTs see individuals when they are symptomatic and may have more time with the patient than physicians do—can develop rapport	• Screen for injuries that might indicate DV • Follow APTA guidelines for supporting DV survivors using a patient-focused approach. • Use an understanding, non-blaming approach • Acknowledge nothing the survivor does justifies the abuser’s violence • Maintain connections at local shelters to consult with and refer patients to	• Many DV shelters can provide in-service training to PT practitioners • Education for PT students should include learning about state laws • Information pamphlets in waiting room
Kessler (2012) USA (Q&A Editorial) *OT, IPV* *Support*	• Support survivors with work performance, coping skills, money management, parenting skills, self-confidence, concentration, stress management, etc. • Cognitive-behavioral approach to increase insight and support in problem-solving	• Client-centered and occupation-based evaluation and assessment • Interventions should emphasize building self-esteem and empowerment • Collaborate with other professionals to support survivors and provide interventions for abusers • Promote a nonjudgmental and safe environment to allow the opportunity for patients to self-disclose	
Koch (2001) USA *OT, IPV (victim advocacy)*	Offer safety, support, and resources to someone seeking refuge from violence and intimidation	• Assess risk for harm • Intervention may also include encouraging and facilitating participation in occupations once denied the victim	• Therapists must educate themselves about the cycle of abuse, its effects on occupation and identity, and how to safely empower survivors • OT must assist in constructing a sensitive, appropriate, and coordinated response that asks each survivor what she needs to repair the harm of abuse • Each situation requires a comprehensive initial assessment of the victim's safety, measuring the risk of harm with available resources and support
**Mandozzi** n.d. USA *OT, IPV* *Support*	• OTs can support survivors of DV through education, also go into schools and juvenile centers to educate individuals on healthy relationships • OTs are very well-versed in considering family dynamics, role fulfillment, routine, and emotional stability, which will be critical in handling DV cases	• OTs required to work with individuals who have sustained brain injuries • Can work within interprofessional teams; can influence legislative changes	• OT services can be implemented in clinics, in support group settings, in a women and children’s shelters, in homes, and with individuals or family members. There are so many ways that OT can help prevent, advocate for, treat, and educate about DV • Speaks about the potential role of OT in supporting DV survivors/victims; further research on their actual role in supporting this population
Olivas-De La O (2008) USA (Perspective/Recommendations) *OT, IPV* *Support*	Prevention, intervention, and recovery of DV are done through educating, volunteering, and making OT a priority in the recovery of survivors.	• Work with the [DV organization's] counselor, social worker, and psychologist on goal setting for clients. • Be the client’s advocate on all levels • Assist the survivor to address life skills for herself and her family (e.g., cooking together with her children, reading) and encourage participation by all those who live with her • Reinforce leisure skills. Encourage survivors to take time outside of therapy to do fun things that are inexpensive but fulfilling	• Demonstrate OTs understanding of participation and occupational performance for self-empowerment of work, education, social participation, leisure, and ADLs • Help influence public policy and ensure that there is funding for OT to be an active part of DV recovery • Collaborate with safe houses or group hands to address communication, occupations, and self-empowerment
Royal College of SLP (2020) England (Response to Tabled Bill) *SLP, IPV* *Support*	SLP have an important role to play in safeguarding vulnerable individuals of all ages		• Identification of communication needs is critical to ensuring individuals impacted by DV can access and fully participate in programs designed to support them
Stancliff (1997) USA (OT Practice Magazine) *OT, IPV* *Screening* *Support*	Survivors may share things with an OT that they would not share with others, OTs need to know what to do in those instances	• Be aware of indicators of possible DV (central pattern of injuries, inconsistent explanations of injuries, multiple injuries in various stages of healing) • Have referral information to provide as needed • Create a supportive, nonjudgmental space for disclosure, and ask about social supports	• Provide information about state laws regarding DV
Thompson et al. (2020) USA (Continuing Education Article) *OT, ST*	To explore important skills that OTs possess for developing interventions, education, research, and advocacy efforts to positively affect the occupational participation of persons who have incurred biopsychosocial deficits because of human trafficking situations	• Occupation-based intervention and client-centered approaches in social participation, physical activity, IADLs, work, education, sleep, and rest used with persons incurring mental health conditions can enhance life satisfaction, emotional stability, community integration, and healthy life roles for trafficking survivors • The 3 Ps: Prevention, Protection, and Prosecution. • OT role in prevention is largely education along with research and offering expertise in the occupations of work, vocational training, education, social participation, and rest	• Develop research methods to assess approaches • Cultivate partnerships among individuals, agencies, healthcare facilities, legislators, public officials, schools, law enforcement, etc. for optimal service delivery • Disseminate outcomes through publications and presentations in OT venues and in diverse fields • Advocate to professional groups, funding sources, and legislative officials and for legislative changes • Develop education, intervention, prevention, and research initiatives that conserve the unique aspect of the occupation • Elaborate services to meet the unique needs of LGBTQ members • Collaborate within and across cultures and national borders

*Note*. ADL = activities of daily living; AOTA = American Occupational Therapy Association; APTA = American Physical Therapy Association; DV = domestic violence; HELP = Health Enhancement Lifestyle Profile; IADL = instrumental activities of daily living; IPV = intimate partner violence; JCAHO = Joint Commission on Accreditation of Healthcare Organizations; MOHO = Model of Human Occupation; OT = occupational therapist; PT = physical therapist; SLP = speech-language pathologist; ST = sex trafficking.

### Identification of Brain Injury

As brain injury, disability, and IPV are highly interrelated and rehabilitation professionals can play a key role in supporting individuals with any or all of these experiences, we additionally reviewed each article to determine whether brain injury was considered. Across categories, there were six studies that focused on women living with disabilities (Ballan & Freyer, 2019, 2020, 2021; Ballan et al., 2021; Gutman et al., 2004; Smith & Strauser, 2008); however, only three specifically discussed brain injury (Ballan & Freyer, 2019; Ballan et al., 2021; Gutman et al., 2004). One additional study, which looked at language functioning among women living in shelters, assessed for brain injury (O’Neil-Pirozzi, 2003). The four articles discussing brain injury are reviewed in the context of their categories below.

Among the intervention articles, only one of eight (13%) mentioned diagnosed or suspected brain injury, noting 19% of the women experiencing domestic violence had sustained a traumatic brain injury substantial enough to receive a diagnosis and medical treatment (Gutman et al., 2004). The authors noted an additional group of women who experienced cognitive impairment with no known cause, which the authors suggest might be a result of the impacts of domestic abuse over time (Gutman et al., 2004). None of the seven articles discussing knowledge or attitudes referred to brain injury or its possible intersections with IPV. Three of the 30 opportunities articles (10%) mentioned brain injury in some way. Two of the primary research articles assessed for brain injury. One identified 25% of their participants screened positive for a possible brain injury using the HELPS screening tool. Participants who screened positive for brain injury were more likely to report difficulty participating in life roles and poorer emotional or cognitive functioning than those who did not (Ballan et al., 2021). In the other primary research article assessing brain injury, authors identified 16% of their sample had experienced at least one head injury, noting that some of the language deficits seen in these individuals may be due to the head injury (O’Neil-Pirozzi, 2003). Finally, one study recommended SLPs assess for brain injury using the HELPS screening tool (Ballan & Freyer, 2019).

## Discussion

This systematic scoping review identified 44 articles across peer-reviewed and gray literature addressing knowledge and awareness of and interventions for IPV among rehabilitation professionals. Articles broadly grouped into three categories: rehabilitation interventions for IPV survivors, assessment of rehabilitation professionals’ knowledge of and attitudes toward IPV survivors, and identified opportunities for rehabilitation professionals to support IPV survivors. The eight studies exploring rehabilitation interventions assessed a highly heterogeneous group of predominantly OT interventions that were highly individualized and designed to support life skills and meaningful engagement. Despite the wide range of intervention approaches, each reported high satisfaction among survivors, functional improvements, or both, indicating a highly tailored approach is beneficial and integral to appropriate IPV support. Assessments of knowledge and attitudes explored a broader range of rehabilitation professionals, with three of the four groups of interest (OT, PT, and physiatry) included. Across professions, there was a noted lack of education about IPV, with rehabilitation professionals often reporting feeling underprepared to support survivors. Rehabilitation professionals expressed a desire for more information and training about IPV as well as protocols for identification. The need for education was further emphasized across the articles discussing opportunities, both within training programs and in continuing education initiatives. This included education on adequately identifying survivors of IPV, developing in-depth safety planning, and ensuring that survivors are referred to necessary supports within the community. Several recommendations for practice change were presented in opportunities articles. First, the importance of a nonjudgmental and trauma-informed approach to supporting survivors was emphasized. Articles further suggested that practitioners or the institutions in which they work should be aware of local resources to which they can refer survivors and, whenever possible, strive for collaboration with other disciplines to support holistic care for survivors. Critically, articles emphasized a client-centered approach to service delivery, involving survivors in all aspects of their care planning (Ballan & Freyer, 2019).

Despite increased IPV among individuals living with disability and heightened risk for disability, particularly brain injury, among IPV survivors, only a small minority of studies addressed disability (*n* = 6, 14%) and/or brain injury (*n* = 4, 9%), all but one of which focused on opportunities for intervention. Notably, none of the articles assessing knowledge and attitudes of providers considered brain injury or disability, preventing an assessment of how the intersecting stigma may impact the way survivors living with a disability may be perceived. Women living with disabilities experience increased rates of all forms of abuse compared to women living without disabilities and can experience “disability-specific abuse” such as neglecting to provide personal care or tampering with assistive devices (Austin et al., 2014). This disability-specific abuse and other challenges such as the abuser being a primary caregiver can hinder the identification of IPV among individuals living with disabilities (Plummer & Findley, 2012). The use of tailored screening tools, such as the Abuse Assessment Screen for disability, can be incorporated to better support IPV identification among individuals living with a disability. Physical abuse sustained during IPV often targets the head and neck region, increasing the likelihood that survivors may sustain a brain injury, which can lead to disability (Gutman, 2021; Sheridan & Nash, 2007). Rehabilitation professionals must be aware of possible brain injuries in IPV survivors to provide appropriate and tailored support. Therefore, education should also include how and when brain injury screening tools should be used (Ballan & Freyer, 2019).

In many instances, rehabilitation professionals are the first point of contact for survivors of IPV and were identified as well positioned to screen for and identify survivors (Dalton, 2005). Providing adequate screening and intervention is crucial to ensure survivors are receiving the support that they need. Furthermore, it is imperative that education and training among rehabilitation professionals and within organizations reflect the complex nature of IPV and includes awareness and specified screening tools for survivors of IPV, including survivors with disabilities and/or brain injury, to ensure that widespread and inclusive support is provided. Several promising practices were identified to assist rehabilitation professionals in supporting IPV survivors including building flexible programs that can be tailored to a survivor’s specific needs, building IPV education into rehabilitation training for professionals, and developing local networks of resources to which they can refer survivors. This review affirmed the dearth of brain injury-sensitive rehabilitation services available to IPV survivors with very few rehabilitation professionals screening or otherwise accounting for brain injury in the included studies. This is especially noteworthy, as many psychological and physiological issues found in IPV survivors have been traditionally associated with the severity of abuse or posttraumatic stress disorder when these challenges may instead be a result of sustaining one or multiple brain injuries (Banks, 2007; Kwako et al., 2011). There is a pressing need for further education, training, and awareness on brain injury considerations in IPV as well as survivors of IPV with disabilities among rehabilitation professionals.

### Recommendations

The need for increased awareness and opportunities for education is a well-documented recommendation for service providers supporting IPV survivors (e.g., Goicolea et al., 2015; Haag et al., 2019; Monahan & O’Leary, 1999; St Ivany & Schminkey, 2016; Toccalino, Haag, Estrella, Cowle, Fuselli, Ellis, Colantonio, 2022). While this need is echoed in this review, we recognize putting this burden on individual providers, on top of the demand of their existing duties, is unlikely to affect systemic change. We therefore propose three system-level changes to better support rehabilitation professionals throughout their training and careers. First, we call on educational institutions to review and revise their curricula to ensure all rehabilitation professionals graduate with at least some awareness of IPV and its connection with brain injury. While we recognize training programs already cover a large volume of content, IPV can be woven into existing aspects of curricula, for example, when learning about disability. Second, we encourage accreditation bodies responsible for continuing education credits to include and promote offerings covering IPV. Most accreditation bodies require a minimum number of continuing education credits on an annual or bi-annual basis. Promoting content that includes or focuses on IPV is one way to support professionals in the workforce gain awareness and skills to support survivors. Finally, we call for cross-sectoral collaboration both within healthcare and between healthcare and IPV services. For organizations such as hospitals or community health clinics who already work in multidisciplinary teams, we encourage engagement with local women’s shelters and IPV services both to support rehabilitation professionals in better supporting IPV survivors and to provide IPV service providers places to refer survivors for rehabilitation-related support. This type of engagement can also be pursued at the individual level, with rehabilitation professionals developing their own networks of other healthcare and IPV service providers. Critical to this collaboration is adequate funding to support the time necessary to develop and maintain these relationships (Toccalino, Haag, Estrella, Cowle, Fuselli, Ellis, Gargaro, et al., 2022).

We further would encourage individual rehabilitation professionals to consider the possibility of IPV among the individuals they support who are living with disability and/or who have experienced brain injury. For rehabilitation professionals already working with IPV survivors, we would encourage them to consider the possibility of brain injury. Finally, we challenge rehabilitation professionals to put aside any assumptions they might have about who experiences IPV—individuals across genders, ethnicities, and economic or social statuses experience IPV.

### Future Directions

Through this review, we identified several promising directions for future research. Very few of the included studies reported on race or ethnicity among study participants and none of the included articles discussed the potential impacts of racism, racialization, or culture, either among survivors or rehabilitation professionals. A recent scoping review investigating integrated care pathways for Black persons reported differential access to and lower quality rehabilitation care for Black people than white people in several included studies (Omar et al., 2023). The authors of studies included in the review discussed systemic inequalities, racial prejudices, and biases as contributors to this differential access, factors that Omar et al. (2023) note describe anti-Black racism. Recognizing, naming, and understanding the impacts of anti-Black, anti-Indigenous, and other forms of racism on IPV survivors seeking rehabilitation are critical to dismantling systems of oppression and providing equitable care.

All articles identified in this review focused exclusively or predominantly on women or female survivors, in most cases with men or males as perpetrators of the abuse. Though some studies noted that men and gender-diverse individuals experience IPV and that IPV can occur outside of heterosexual relationships, none specifically explored rehabilitation among these groups, indicating an opportunity for future research.

While the volume of articles discussing opportunities for rehabilitation professionals to support survivors indicates an awareness of IPV, only a small subset of the articles in this review discussed specific interventions for IPV survivors with very little overlap in assessment tools or approaches, suggesting more research is required to determine what works for survivors and under what circumstances. Furthermore, only a small subset of articles explored the knowledge and attitudes of rehabilitation professionals, most of which were published more than 15 years ago, before the acknowledgment of IPV as a public health crisis. More investigation into what rehabilitation professionals know and believe about IPV and how training programs can better support them in supporting survivors is warranted, both broadly and in the more specific contexts of disability and brain injury.

Finally, the overwhelming majority of literature included in this review centered on OTs as a profession. Future work to support PTs, SLPs, and physiatrists in their care of IPV survivors, as well as more work on how these professions can work together in supporting survivors would be beneficial. Encouragingly, we are aware of a forthcoming resource for SLPs on supporting IPV survivors, suggesting that awareness is growing (Wiseman-Hakes et al., In Press).

#### Strengths and Limitations

To our knowledge, this review is the first systematically conducted, peer-reviewed synthesis of the literature exploring the role of rehabilitation professionals for survivors of IPV and sex trafficking. Our search strategy spanned 10 databases and included a search of gray literature, increasing the breadth of article types identified. Furthermore, there were no date, language, or geographic restrictions placed on the search. Translation services were used to ensure articles published in languages other than English were not automatically excluded and to broaden the scope of included articles.

As with any review, the findings presented here are limited by the literature included. Most of the primary research articles had small or convenience samples and there was little overlap in the assessments used across studies, limiting the ability of this review to compare findings or identify best or promising practices. Furthermore, many of the included articles were published more than 15 years ago, before the acknowledgment of IPV as a public health crisis. Finally, 86% of the articles included were conducted in the United States, and 68% were centered on OTs, which may limit the generalizability of findings to other health systems, professions, or settings.

## Conclusions

PTs, OTs, SLPs, and physiatrists are highly likely to encounter survivors of IPV and sex trafficking in their practice and are in a prime position to provide support and care. Given this opportunity, it is imperative that in addition to being well-placed, these professionals are well equipped to aid in IPV survivor rehabilitation. Despite the identifiable gaps in the literature, in research, and practice concerning IPV survivors, vital information was gathered on roles that rehabilitation professionals presently engage in when supporting IPV. This review suggests numerous recommendations that rehabilitation professionals can utilize to ensure that IPV-specified programs or supports are incorporated within their practices.

## Critical Findings

1. There is both an opportunity and a need for rehabilitation professionals to support IPV survivors.
2. Generally, rehabilitation professionals report being underprepared to support IPV survivors; they require more education and training to confront biases and be better able to identify and support survivors.
3. Interventions for IPV survivors to support life skills, activities of daily living, and meaningful engagement are positively received and result in improvements in the areas being addressed.

## Implications for Practice, Policy, and Research

**Table:** 

Practice	• Rehabilitation professionals should seek education and training opportunities to learn more about identifying IPV and how to support survivors within their practice. • Local resource networks should be developed and maintained so IPV survivors can be referred to appropriate support. • Rehabilitation programs should be flexible and tailored to survivors’ specific needs.
Policy	• IPV training should be embedded into curricula and organizational policies for rehabilitation professionals.
Research	• Further investigation is needed into appropriate rehabilitation interventions for IPV survivors as well as education and training supports for rehabilitation professionals.

## Supplemental Material

sj-docx-1-tva-10.1177_15248380231196807 – Supplemental material for Exploring the Relationships Between Rehabilitation and Survivors of Intimate Partner Violence: A Scoping Review Supplemental material, sj-docx-1-tva-10.1177_15248380231196807 for Exploring the Relationships Between Rehabilitation and Survivors of Intimate Partner Violence: A Scoping Review by Danielle Toccalino, Gifty Asare, Jenna Fleming, Joyce Yin, Amy Kieftenburg, Amy Moore, Halina (Lin) Haag, Vincy Chan, Jessica Babineau, Nneka MacGregor and Angela Colantonio in Trauma, Violence, & Abuse

sj-docx-2-tva-10.1177_15248380231196807 – Supplemental material for Exploring the Relationships Between Rehabilitation and Survivors of Intimate Partner Violence: A Scoping Review Supplemental material, sj-docx-2-tva-10.1177_15248380231196807 for Exploring the Relationships Between Rehabilitation and Survivors of Intimate Partner Violence: A Scoping Review by Danielle Toccalino, Gifty Asare, Jenna Fleming, Joyce Yin, Amy Kieftenburg, Amy Moore, Halina (Lin) Haag, Vincy Chan, Jessica Babineau, Nneka MacGregor and Angela Colantonio in Trauma, Violence, & Abuse
