# Supplementary material for: Exploring the Relationships Between Rehabilitation and Survivors of Intimate Partner Violence: A Scoping Review
Source: Trauma Violence Abuse. 2023 Sep 30;25(2):1638–60. doi: 10.1177/15248380231196807 (PMC10913349; doi:10.1177/15248380231196807)
Supplement: sj-docx-1-tva-10.1177_15248380231196807 – Supplemental material for Exploring the Relationships Between Rehabilitation and Survivors of Intimate Partner Violence: A Scoping Review [file sj-docx-1-tva-10.1177_15248380231196807.docx]

# Appendix I: Search Strategy

## Databases searched:

- MEDLINE(R) ALL (Ovid)
- Cochrane Central Register of Controlled Trials (Ovid)
- Embase Classic + Embase (Ovid)
- APA PsycInfo (Ovid)
- CINAHL Complete (EBSCO)
- Criminal Justice Abstracts (EBSCO)
- Nursing and Allied Health Premium (Proquest)
- Applied Social Sciences Index & Abstracts (Proquest)
- Dissertations and Theses Global (Proquest)
- Web of Science Core Collection (Web of Science)

## MEDLINE(Ovid) Search Strategy

1. "Physical and Rehabilitation Medicine"/
2. exp rehabilitation/
3. rehab*.tw,kf,jw.
4. telerehab*.tw,kf,jw.
5. neurorehab*.tw,kf,jw.
6. rh.fs.
7. Rehabilitation Centers/
8. (physiatrist? or physiatry).tw,kf.
9. occupational therapy/
10. (occupational adj therap*).tw,kf,jw.
11. physical therapy specialty/
12. (physical adj therap*).tw,kf,jw.
13. physiotherap*.tw,kf,jw.
14. physio-therapist*.tw,kf,jw.
15. Speech-Language Pathology/
16. (speech adj2 (therap* or patholog*)).tw,kf,jw.
17. or/1-16
18. Domestic Violence/ or (domestic adj3 (abus* or violen*)).tw,kf.
19. exp Intimate Partner Violence/ or Battered Women/ or ((partner* or spous* or wife or wives or marital or couple* or husband* or boyfriend* or girlfriend*) adj3 (abus* or violen* or aggression or batter*)).tw,kf.
20. Sex work/ or (sex work).tw,kf. or (sex adj3 industr*).tw,kf. or (prostitut*).tw,kf. or (sex* adj1 transact*).tw,kf.
21. 18 or 19 or 20
22. 20 and 24
23. 25 not (exp animals/ not exp humans/)
