# Supplementary material for: Exploring the Relationships Between Rehabilitation and Survivors of Intimate Partner Violence: A Scoping Review
Source: Trauma Violence Abuse. 2023 Sep 30;25(2):1638–60. doi: 10.1177/15248380231196807 (PMC10913349; doi:10.1177/15248380231196807)
Supplement: sj-docx-2-tva-10.1177_15248380231196807 – Supplemental material for Exploring the Relationships Between Rehabilitation and Survivors of Intimate Partner Violence: A Scoping Review [file sj-docx-2-tva-10.1177_15248380231196807.docx]

# Appendix II: Tools Used Across Included Studies

## Occupational Therapy Assessments from Intervention Articles

- Adolescent/Adult Sensory Profile (AASP)
  - Cerny et al. 2019
- Sensory Modulation Screening Tool
  - Cerny et al. 2019
- Canadian Occupational Performance Measure (COPM)
  - Cerny et al. 2019
  - Mangum et al. 2019
  - Clarke & Jones 2021
- Goal attainment scales (individually tailored)
  - Gutman et al. 2004
- Executive Functional Performance Test (EFPT)
  - Mangum et al. 2019
- Occupational Therapy Task Observation Scale (OTTOS)
  - Mangum et al. 2019
- Occupational Performance History Interview (OPHI-II)
  - Fitzgerald et al. 2017
  - Helfrich & Rivera 2006
- Rosenburg Self-Esteem Scale (RSES)
  - Fitzgerald et al. 2017
- Quality of Life Rating (QOLR)
  - Fitzgerald et al. 2017
- Occupational Circumstances Assessment Interview Rating Scale (OCAIRS)
  - Clarke & Jones 2021
- Occupational Self-Assessment (OSA)
  - Helfrich & Rivera 2006
- Assessment of Motor and Process Skills (AMPS)
  - Helfrich & Rivera 2006
- Assessment of Communication and Interaction Skills (ACIS)
  - Helfrich & Rivera 2006

## Knowledge/Beliefs/Attitudes Assessments

- Experiences and Attitudes towards Family violence (Unnamed Survey)
  - Williamson et al., 2004; Johnston et al., 2001)
- Domestic Violence Questionnaire (modified)
  - Shahgangar et al., 2004
- Physician Readiness to Manage Intimate Partner Violence Survey (PREMIS) adaptation
  - Sivagurunathan et al., 2019
- Health Care Provider Survey for Domestic Violence
  - Sivagurunathan et al., 2019

## Assessments recommended in articles exploring Opportunities

- HELPS Screening tool
  - Ballan et al., 2021
- Canadian Occupational Performance Measure (COPM)
  - Javaherian-Dysinger et al., 2016
- Community-based Practice Evaluation
  - Javaherian-Dysinger et al., 2016
- Kawa Model to support survivors of DV
  - Humbert et al., 2014
- Occupational profile
  - Javaherian-Dysinger et al. 2017
- Community-Based Occupational Therapy Evaluation
  - Javaherian-Dysinger et al. 2017
- Health Enhancement Lifestyle Profile (HELP)
  - Javaherian-Dysinger et al. 2017
- Adult Sensory Profile
  - Javaherian-Dysinger et al. 2017
- IPV Screening Tool for Physical Therapists
  - Ballan & Freyer 2021
- Abuse Assessment Screen - Disability
  - Ballan & Freyer 2021
